# Self-assessed digital competence of nurse educators—A cross-sectional study in four countries

**DOI:** 10.1177/20552076251395451

**Published:** 2025-11-10

**Authors:** Juha Pajari, Marjorita Sormunen, Leena Salminen, Imane Elonen, Miko Pasanen, Michelle Camilleri, Laia Wennberg-Capellades, Elaine Haycock-Stuart, Andrea Sollárová, Terhi Saaranen

**Affiliations:** 1 Department of Nursing Science, Faculty of Health Sciences, 4344 University of Eastern Finland, Kuopio, Finland; 2 Faculty of Health Sciences, Institute of Public Health and Clinical Nutrition, 4344 University of Eastern Finland, Kuopio, Finland; 3 Department of Nursing Science, 8058 University of Turku, Turku, Finland; 4 Turku University Hospital, Turku, Finland; 5 Turku University of Applied Sciences, Turku, Finland; 6 Department of Nursing, Faculty of Health Sciences, 37563 University of Malta, Msida, Malta; 7 Nursing Department, School of Medicine and Health Sciences, 16760 Universitat Internacional de Catalunya, Barcelona, Spain; 8 Nursing Studies, School of Health in Social Science, 523476 University of Edinburgh. Elsie Inglis Quad, The Medical School, Edinburgh, Scotland; 9 Department of Nursing, Faculty of Social Sciences and Health Care, 112841 Constantine the Philosopher University in Nitra, Nitra, Slovakia

**Keywords:** Competence, digital technology, health professions education, nursing faculty, teacher, teaching

## Abstract

**Objective:**

This study examines the self-assessed level of nurse educators’ digital competence in four European countries and explores variables associated with it.

**Design:**

A cross-sectional study was conducted.

**Method:**

Nurse educators (n = 290) from 36 nursing education organizations in Finland, Malta, Slovakia, and Spain participated in the study. Data were collected from May 2021 to February 2022 through an online survey. The Educators and Educator Candidates’ Competence in Digital Pedagogy instrument contained 20 items in three categories. Descriptive statistics, the Kruskal–Wallis test, one-way analysis of variance (ANOVA), and multiple regression analysis were used in the analysis.

**Results:**

Nurse educators reported an overall moderate level of digital competence; the lowest level was in the safe and responsible use of technology. Slovak educators assessed their competence as higher than those from Finland, Malta, and Spain. Having a master's or doctoral degree and completing pedagogical studies were related to a higher level of digital competence.

**Conclusion:**

Nurse educators have successfully adopted the use of evolving teaching, learning, and assessment methods to ensure appropriate and practice-preparing healthcare education. The findings can be used in nursing education and healthcare practice organizations to focus nurse educators’ education on the safe and responsible use of digital pedagogical practices, such as the ethical utilization of electronic documents. Furthermore, in-depth examination of the relationship between educators’ education and digital competence is needed.

## Introduction

The increased use of technology has emphasized the need for educational organizations and educators to update their readiness to implement high-quality education utilizing digital pedagogical practices.^1,2^ As highlighted in the publication *Essentials: Core Competencies for Professional Nursing Education*,^3^ technology is increasingly integrated into nursing education. To meet these reforming competence requirements of the changing educational landscape, nurse educators may need to become more accustomed to new working methods.^4^

There is currently a diversity of technology within educational organizations, for example, an increased amount of open online learning materials are available to everyone.^5^ Along with this utilization of technology, there are promising examples that technology enables access to knowledge and information, enriching educational processes and improving students’ learning outcomes.^6–9^ In addition, incorporating technology into nursing education prepares students to utilize technology, which can later positively impact patient outcomes. Examples of this positive value include engaging the patient and using digital technology for supporting the nurse–patient relationship with communication^3^ and enabling creative nurse-driven solutions to complex healthcare challenges.^10^

However, there has been concern that technology can heighten learning inequality, increase student isolation, narrow educational experiences, and violate privacy.^5^ For this reason, it is necessary to ensure that nurse educators have the required digital competence to implement modern healthcare education and to teach digital practices to their students to achieve the positive outcomes.^11–13^ It is important to examine the digital competence of nurse educators to understand better and develop education to meet their specific needs so that they can provide a high-quality learning environment for their students.

The digital competence of a nurse educator has yet to be unanimously defined.^14^ In this study, digital competence is understood by the definition of the European Commission, whereby digital competence is the confidential and critical utilization of information technology in various actions.^15^ Additionally, this study refers to the view of the European Framework for the Digital Competence of Educators (DigCompEdu framework), where an educator's digital competence is seen as consisting of six competence areas, including 22 competence descriptions (Supplemental material 1, Figure 1).^16^ Moreover, this study describes the digital competence of nurse educators in their educational work in nursing education organizations. Technology is thus understood as educational technology, including devices (e.g. computers and mobile phones) and resources (e.g. applications and digital materials, such as games, learning environments or text and video files).^16^

This DigCompEdu framework and other definitions of digital competence have in common that they describe how technology is utilized in a versatile way in the digital pedagogical practices, enabling communicative teaching, learning, and assessment methods.^14^ Skantz-Åberg et al.^17^ noted that the DigCompEdu framework is, in recent studies, one of the most used when referring to educators’ digital competence. However, there may be a need to continuously redefine the framework's content due to rapid technological development.^17^ For example, this development can be seen in the potential of learning analytics to collect study data and allocate support for special learning needs.^2,18^ Further, the potential of the use of artificial intelligence (AI) as ChatGPT (Chat Generative Pre-Trained Transformer) to create material, such as text, images, and audio, for rapidly changing teaching and learning processes needs to be considered.^2,19^

Educators’ digital competence seems to have been studied recently in contexts such as primary or secondary education^20^ and varying disciplines, such as business sciences.^21^ While Santos et al.^14^ stated that research on the digital competence of educators in higher education is still at an early stage, some recent studies focus on the digital competence of nurse educators or educators working in healthcare education. For example, several studies published in Europe^22–26^ and in Asia^27,28^ have explored this topic. In these studies, the digital competence of healthcare educators has been seen to be at a basic to intermediate level. However, there are several needs regarding readiness for online teaching that need addressing for the future enhancements of continuous education. Despite the evidence about the deficiencies in healthcare educators’ digital competence, it is promising that educators are versatile in their use of digital methods and are interested in developing further their competence.^23^ Furthermore, continuous education targeted at digital teaching and learning has been linked to improved digital competence of healthcare educators.^24^ Additionally, nurse educators have seen it essential to develop their digital competence in a digitalized healthcare education environment.^29,30^

Some previous studies have examined digital competence and the relationship with the nurse educators’ backgrounds, but research still needs to be conducted. For example, a younger age in educators is related to better digital competence^6,28^ and a more versatile use of digital technology in the educator's work.^23^ In addition, the professional category of junior lecturers, possibly including younger educators, has been identified to have greater digital competence.^26^ In previous studies, where the nurse or healthcare educators’ competence has been examined more broadly than digital competence, the level of education, experience, and completed pedagogical studies have been positively related to the educator's competence.^31,32^ Examining these background variables makes it possible to target education for specific groups of nurse educators if differences in competence between them are revealed. However, more research is needed on the relationship between nurse educators’ background variables and their digital competence.

To summarize, only a few recent studies have examined the digital competence of nurse educators or educators in healthcare education. More comparative research is needed to clarify and identify the current state of educators’ digital competence. Based on the evidence, educators’ basic and continuous education could be enhanced for greater digital competence.^11,12^^32–34^

This study was part of a wider Erasmus + funded the New Nurse Educator project, whose purpose was to study, develop, and harmonize the education of nurse educators in Europe. The partners were universities from five European countries (Finland, Malta, Scotland, Spain, and Slovakia). This substudy focuses on the digital competence of nurse educators in four European countries (Finland, Malta, Spain, and Slovakia). It examined nurse educators’ digital competence level and explored the educators’ background variables that explain digital competence to produce knowledge of the competence development needs. The research questions were:

1. What is the level of digital competence of nurse educators in the four European countries?
2. What is the relationship between nurse educators’ background variables and the educators’ digital competence in four European countries?

## Materials and methods

### Study context and participants

The target group of this cross-sectional study was nurse educators from higher education nursing education organizations from four countries: Finland, Malta, Slovakia, and Spain. Across these countries, there is heterogeneity in the education requirements for nurse educators. For example, in these countries, qualified nurse educators must have either a master's degree (Finland, Malta, and Slovakia) or a doctoral degree (Spain) in health sciences. In Malta, a master's degree can also focus on pedagogy, anthropology, or sociology. In these four countries, pedagogical studies are not mandatory but may be included in the degree, as in Finland.^35^ As the definition of a nurse educator and the educators’ educational requirements vary in Europe, in this study, the nurse educator is assumed to have the competence to utilize various evidence-based methods in planning, implementing, and evaluating nursing education. In this way, educators can teach theoretical and practical skills in nursing education organizations aimed at a nurse's degree.^35^

### Instrumentation

The instrument used for data collection was the Educators and Educator Candidates’ Competence in Digital Pedagogy (Finnish: Opettajien ja Opettajaopiskelijoiden DIgipedagoginen osaaminen (OODI)) scale, based on the DigCompEdu framework.^16^ The OODI was developed^24^ and validated^36^ in Finland, where the participants were nurse educators. The instrument includes two visual analog scale (VAS) items for assessing educators’ digital competence and interest in educational technology. The scale for these items ranges from 0 (weak) to 10 (strong). Moreover, the OODI contains 20 items scored on a Likert scale, which reflects competence (1 = not at all, 5 = very well). These items are divided into three factors. In the Finnish context, high reliability is evidenced by a Cronbach's α value of 0.938 and McDonald's ω value of 0.940; factor values ranged between 0.833 and 0.889 for α and 0.885 and 0.902 for ω. The OODI was formulated in Finnish and translated into English.^36^ In this study, the OODI was also translated into Spanish and Slovak and back-translated into English. Independent translators undertook the translation. The final back-translation was then translated into Finnish, and the research group evaluated the translation's consistency. Nurse educators pretested the OODI in Spanish and Slovak to ensure the translations’ comprehensibility.^37,38^

### Data collection

The data were collected as part of the New Nurse Educator project between May 2021 and February 2022. The sample size was based on a power analysis conducted for the entire dataset for the New Nurse Educator project. The project partners, guided by a statistician, estimated the number of nursing education organizations to be recruited to achieve a sufficient sample. Cluster sampling was used, and organizations were selected based on a European nomenclature of territorial units for statistics (French: Nomenclature des Unités Territoriales Statistiques (NUTS)) classification.^39^ The basic regions of NUTS 2 in Finland and Slovakia and NUTS 1 in Spain were applied. Total sampling was used in Malta due to the country's relatively small size. The target organizations were chosen to evenly cover the NUTS regions. The organizations’ inclusion criteria were that they offered a minimum of bachelor's-level education in nursing. In practice, the organizations in Finland were universities of applied sciences, and in Malta, Spain, and Slovakia, they were universities.

The project coordinator from Finland was responsible for data collection and implemented the various language versions of the OODI instrument to the online survey in the REDCap (the Research Electronic Data Capture) software (Vanderbilt University, Nashville, Tennessee). The survey of the whole project contains three instruments, the OODI instrument being the last section of the main survey. A representative of the project partners in each of the four European countries contacted and sent an online survey (in Finnish, Spanish, Slovak, or English in Malta) to contact persons (e.g. the head of nursing education) of participating nursing education organizations (total N = 36, Finland: 9, Spain: 17, Slovakia: 9, Malta: 1). The organizations’ contact persons emailed the survey to nurse educators (N = 1163) in their respective countries. The inclusion criterion for educators was working as a nurse educator in the target organization. The exclusion criterion was only working in a hospital or other clinical setting as a clinical instructor. A reminder to participate in the study was sent three times. Data from the four countries’ nurse educator respondents formed one main dataset in the REDCap software.

### Data analysis

Data were analyzed using R Statistical Software version 4.0.2.^40^ First, the data were screened for missing values. When there were only minor missing values (< 5%) for individual participants in random items of the OODI instrument, all available data were included in the analyses.^41,42^ Cronbach's α and McDonald's ω coefficients were analyzed to verify the OODI's internal consistency. Means and standard deviations were examined from the data, and frequencies and percentages were also used in the description. The normality assumption was examined through residuals of analysis of variance (ANOVA), conducting visual inspection and Shapiro–Wilk tests with Levene's test. For VAS items, the histograms and the Shapiro–Wilk test showed non-normality (p < 0.001). Therefore, the Kruskal–Wallis test was used for VAS items to test the educators’ digital competence and interest in educational technology between countries. For the mean of OODI Likert scale items (“total digital competence”), the histogram and Shapiro–Wilk test showed normality (p = 0.14). For factors of the OODI, the histograms were relatively normal, and the Shapiro–Wilk test showed non-normality (Factor 1: p = 0.02, Factor 2: p = 0.04, Factor 3: p = 0.02); however, according to Levene's test, the variances were equal (Factor 1: p = 0.23, Factor 2: p = 0.70, Factor 3: p = 0.85). It is acknowledged that unequal group sizes can affect the ANOVA's robustness. This concern is mitigated when the homogeneity of variance assumption is met. As Blanca et al.^43^ found, the ANOVA retains acceptable levels of type I error and statistical power under moderate non-normality and unequal group sizes, provided that variances are approximately equal. Based on these analyses, a one-way ANOVA was used for the whole OODI scale and its factors to test educators’ digital competence between countries. The statistically significant differences between several groups were further examined using Dunn's and Tukey's post hoc tests. Multiple regression analysis (Enter method) was used to investigate the relationship between educator's background variables (self-assessment of interest in educational technology, age, highest level of education, clinical work experience, pedagogical studies, participation in continuous professional education, and work experience as an educator) and the self-assessment of digital competence. The highest level of education, pedagogical studies, and participation in continuous professional education variables were formed as binary dummy variables (university degree = master's or doctoral degree / yes = 1, other degree / no = 0). When results were reported, the following competence level description was applied: 1.00–1.99: low, 2.00–2.99: tolerable, 3.00–3.99: moderate, and 4.00–5.00: high. In this study, the level of statistical significance was set at p < 0.05.^44^

### Ethical considerations

*The European Code of Conduct for Research Integrity*^45^ was followed during this study. The Turku University's ethics committee approved the study (16 February 2021). Research permissions from nursing education organizations were granted. All educators were informed about voluntary participation with an information letter about the study and a privacy notice sent by email via contact persons. The collected data were treated anonymously. The original authors granted permission to use and reproduce the OODI instrument.^46^ This study followed *the General Data Protection Regulation (GDPR)*.^47^

## Results

### Participants background

Overall, 290 nurse educators responded to the online survey, resulting in a 25% response rate. The responses included Finland: 38%, Spain: 37%, Slovakia: 18%, and Malta: 7%. The nurse educators’ average age was 48 years (min 24–max 67), and 92% had a university degree (master's or doctoral degree). On average, they had 16 years (min 0–max 42) of clinical work experience in nursing and about 13 years (min 0–max 45) of work experience as an educator. The majority of the educators had completed pedagogical studies (73%) and had participated in continuous professional education (82%) (Supplemental material 2, Table 1).

**Table 1.** Nurse educators’ digital competence and interest in technology as self-assessed with visual analog scales.

Item*	Country Mean (SD)					Used test, p-value	Effect size (ε^2^), 95.0% CI [lower –upper]
	Finland	Malta	Spain	Slovakia	All		
Self-assessment of digital competence	3.87 (0.63)	3.40 (0.93)	3.80 (0.57)	3.96 (0.63)	3.83 (0.64)	† *X*^2^(3) = 9.46, **0.024**	0.03 [0.008–0.094]
Self-assessment of interest in educational technology	4.20 (0.65)	3.90 (0.79)	4.28 (0.52)	4.30 (0.67)	4.23 (0.62)	† *X*^2^(3) = 5.75, 0.124	0.02 [0.003–0.077]

*Visual analog scale; †Kruskal–Wallis test; (ε^2^): Epsilon squared.

CI: confidence interval for effect size; SD: standard deviation.

### Nurse educators’ digital competence

With the VAS, educators in four countries self-assessed their digital competence as moderate (mean 3.83, SD 0.64). Differences (p < .05) emerged in educators’ assessments between the four countries. According to Dunn's test, Slovak educators rated their competence higher (mean 3.96, SD 0.63) than Spanish educators (mean 3.80, SD 0.57). Finnish educators (mean 3.87, SD 0.63) and Slovak educators rated their competence higher than the Maltese educators (mean 3.40, SD 0.93) (p < 0.05). Nurse educators self-assessed their interest in educational technology at a high level (4.23 ± 0.62), without significant differences between countries (Table 1).

Self-assessed with the OODI instrument, nurse educators’ digital competence level was moderate (mean 3.51, SD 0.64) (Table 2). Slovak educators’ self-assessed competence was higher (mean 3.74, SD 0.62) than that of their Maltese colleagues (mean 3.26, SD 0.8) (p < 0.05), the effect size (η² = 0.03, 95% confidence interval (CI) [0.000–0.077]) indicating limited practical significance (Table 2). Among the competence areas, the educators’ digital competence was highest in *implementing appropriate independent and community learning* (mean 3.80, SD 0.63). Digital competence was rated as the lowest for *acting safely and responsibly* (mean 3.36, SD 0.79). Here, a difference emerged between the countries (p < 0.05), but the result of Tukey's test and the effect size (η² = 0.03, 95% CI [0.000–0.067]) suggested that the observed difference is unlikely to be meaningful in practice. Educators’ digital competence in *guiding learning based on the evidence* was self-assessed as moderate level (mean 3.44, SD 0.75). In this area, Slovak educators rated their competence (mean 3.73, SD 0.70) higher than Finnish educators (mean 3.34, SD 0.77) (p < 0.05).

**Table 2.** Nurse educators’ digital competence level as self-assessed with OODI instrument.

OODI instrument and items	Country Mean (SD)					Used test, p-value	Effect size (η²), 95.0% CI [lower -upper]
	Finland	Malta	Spain	Slovakia	Total		
OODI (total digital competence) (α=0.949; ω=0.954)	3.49 (0.61)	3.26 (0.81)	3.48 (0.64)	3.74 (0.62)	3.51 (0.64)	‡ F(3, 259) = 2.952 **0.033**	0.03 [0.000–0.077]
*Implementing appropriate independent and community learning* (α=0.905; ω=0.893)	*3.83* (*0.57)*	*3.56* (*0.75)*	*3.77* (*0.67)*	*3.88* (*0.64)*	*3.80* (*0.63)*	‡ F(3, 272) = 1.185 0.316	0.01 [0.000–0.041]
Using technology to collaborate with colleagues, students, and other partners	4.32 (0.62)	4.05 (0.91)	4.27 (0.78)	4.29 (0.70)	4.28 (0.72)		
Using digital resources to support my continuous professional development	3.76 (0.87)	3.75 (0.85)	4.06 (0.73)	3.94 (1.03)	3.91 (0.85)		
Evaluating of own digital competence	4.09 (0.71)	3.45 (0.76)	3.69 (0.92)	3.75 (0.96)	3.83 (0.86)		
Using technology to guide learners	3.97 (0.73)	3.60 (0.94)	3.70 (0.86)	3.92 (0.85)	3.84 (0.82)		
When choosing digital resources, consider their suitability for teaching and learning	3.76 (0.69)	3.56 (0.62)	3.70 (0.78)	4.00 (0.74)	3.77 (0.73)		
Producing digital learning materials, considering their suitability for teaching and learning	3.59 (0.81)	3.55 (0.94)	3.67 (0.93)	3.84 (0.83)	3.66 (0.87)		
Using technology in teaching to enable collaborative learning	3.65 (0.83)	3.55 (0.89)	3.58 (0.84)	3.78 (0.71)	3.64 (0.82)		
Supporting learners’ self-direction through technology	3.59 (0.82)	3.45 (1.10)	3.58 (0.84)	3.62 (0.81)	3.51 (0.87)		
*Acting safely and responsibly* (α=0.903; ω=0.885)	*3.32* (*0.74)*	*3.09* (*0.9)*	*3.33* (*0.79)*	*3.62* (*0.81)*	*3.36* (*0.79)*	‡ F(3, 278) = 2.643 **0.049**	0.03 [0.000–0.067]
Protecting the privacy of sensitive digital material used in teaching	3.32 (1.10)	3.75 (0.91)	3.77 (1.03)	3.94 (1.00)	3.62 (1.07)		
Using digitally licensed digital materials in the right way	3.45 (1.00)	3.60 (0.94)	3.59 (1.09)	3.84 (1.06)	3.58 (1.04)		
Guiding learners to the critical use of digital media	3.71 (0.84)	2.80 (1.15)	3.54 (0.88)	3.64 (0.96)	3.57 (0.92)		
Supporting learners to produce responsible digital content	3.37 (0.92)	2.95 (1.10)	3.21 (0.99)	3.55 (0.89)	3.31 (0.96)		
Guiding the development of learners’ information literacy in a digital learning environment	3.35 (0.84)	2.70 (1.08)	3.25 (0.90)	3.42 (0.99)	3.28 (0.92)		
Supporting learners’ ability to solve problems with technology	3.14 (0.90)	2.90 (1.12)	3.14 (0.92)	3.35 (0.91)	3.16 (0.93)		
Guiding learners to protect the material they produce in accordance with copyright law	2.88 (1.06)	2.90 (0.97)	2.94 (1.17)	3.58 (1.05)	3.03 (1.12)		
*Guiding learning based on the evidence* (α=0.893; ω=0.890)	*3.34* (*0.77)*	*3.27* (*0.89)*	*3.43* (*0.69)*	*3.73* (*0.70)*	*3.44* (*0.75)*	‡ F(3, 278) = 3.517 **0.016**	0.04 [0.001–0.081]
Using technology to give feedback to learners	3.47 (0.96)	3.50 (1.00)	3.28 (0.89)	3.80 (0.88)	3.55 (0.92)		
Motivating learners in a digital environment	3.51 (0.80)	3.15 (1.09)	3.47 (0.84)	3.70 (0.81)	3.50 (0.84)		
Using technology in individualized teaching	3.33 (0.89)	3.25 (1.02)	3.53 (0.78)	3.80 (0.76)	3.48 (0.85)		
Using technology to assess learning according to different assessment methods	3.47 (0.90)	3.40 (0.99)	3.42 (0.83)	3.64 (0.88)	3.47 (0.87)		
Using learning analytics to assess the progress of learning	2.98 (1.01)	3.05 (1.05)	3.28 (0.89)	3.70 (0.89)	3.22 (0.98)		

‡One-way ANOVA; for amounts of participants (n), see Supplemental material 3, Table 2.

α: Cronbach α; ω: McDonald ω; η²: eta squared; ANOVA: analysis of variance; CI: confidence interval for effect size; SD: standard deviation.

More specifically, in *implementing appropriate independent and community learning*, nurse educators self-assessed their use of technology for collaboration at a high level of competence (mean 4.28, SD 0.72). This competence was consistent across the four countries, and the majority (90%) of educators rated that they could use technology for collaboration well or very well (Supplemental material 3, Table 2).

The lowest levels of competence were assessed in *acting safely and responsibly*. Nurse educators assessed that their lowest individual competence was guiding learners to protect their produced materials per copyright laws (mean 3.03, SD 1.12). Here, 32% of educators rated that they cannot at all or can poorly guide their students to do so.

In *guiding learning based on the evidence*, educators assessed competence in using learning analytics to evaluate learning progress at level 3.22 (SD 0.98). When almost a quarter of nurse educators (22%) in all countries rated that they cannot at all or can poorly use learning analytics in this way.

### Background variables related to educators’ digital competence

The multiple linear regression analysis results show the relationship of educators’ background variables with self-assessed digital competence. Educators’ interest in educational technology was directly related to digital competence, and the model explained 28% of the variance in assessed digital competence. In the second model, in addition to an interest in technology, having completed a university degree and having completed pedagogical studies were directly associated with the self-assessment of one's digital competence. The model explains 32% of the variation in digital competence assessment (Table 3).

**Table 3.** Multiple linear regression models of background variables with educators’ self-assessed digital competence as dependent variable

Linear regression model							95.0% Confidence interval for B	
	B	Standard error	β	t	p		Lower	Upper
Model 1** R: 0.286; adjusted R^2^: 0.284; F(1, 280) = 112.2								
Constant	1.456	0.226		6.431	**< 0.001**		−0.099	0.099
Interest in educational technology*	0.560	0.053	0.560	10.594	**< 0.001**		0.436	0.634
Model 2** R: 0.336; adjusted R^2^: 0.321; F(6, 262) = 22.11								
Constant	1.171	0.337		3.476	**< 0.001**		−1.321	−0.462
Interest in educational technology*	0.544	0.054	0.515	10.042	**< 0.001**		0.414	0.616
Age	−0.008	0.006	−0.121	−1.415	0.158		−0.290	0.047
Highest level of education	0.447	0.126	0.690	3.556	**< 0.001**		0.308	1.072
Clinical work experience	0.005	0.004	0.069	1.060	0.290		−0.059	0.198
Pedagogical studies	0.229	0.079	0.354	2.891	**< 0.001**		0.113	0.596
Work experience as an educator	0.008	0.005	0.115	1.449	0.149		−0.041	0.271

Note: Variable “participation continuous professional education” has been removed from model 2 as it was not significant to the model output.

*Visual analog scale; **p **<** **0.001**VIF: range 1.04–2.89.

B: unstandardized coefficients; β: standardized coefficients; VIF: variance inflation factor.

## Discussion

## Results overview

This study examined nurse educators’ level of digital competence across four European countries. Nurse educators in all four countries self-assessed their digital competence as moderate and reported a high level of interest in educational technology. Previously, educators have also expressed interest in using educational technology.^6,11,21^ Likewise, consistent with the few earlier studies, educators’ digital competence in healthcare education seems to be at an intermediate level.^22–24^^,26^ This study's results confirm the previous finding that with the emergence of new technologies in digitalized education within nursing,^3,29,30^ nurse educators have successfully adopted the use of new technology as part of their work. However, with a paucity of cross-national research on nurse educators’ digital competence, this study produced a few new perspectives.

The highest level of self-assessed competence the nurse educators reported related to using technology for collaboration with colleagues, students, and other partners was particularly noteworthy. Educators’ high level of competence in using technology for collaboration is not surprising given the central role that digital communication channels played during the pandemic in maintaining remote interaction among nurse educators and students.^11,22^ Because the data collection for this study took place after the COVID-19 pandemic, the experiences during the pandemic may be essential for the nurse educators’ self-assessed digital competence. However, in the study by Pramila-Savukoski et al.,^8^ the health sciences students expressed that at least some healthcare educators still need to develop competence in teaching with technology and enabling presence and collaboration. Various methods can address these areas, such as using a camera in meetings and using various digital channels, presentations, and digital materials to increase students’ interest.^8^

Nurse educators self-assessed their competence as least developed in acting safely and responsibly, but their competence still suggests a moderate level of attainment. This competence area includes competences such as guiding learners to protect produced materials in accordance with copyright laws and guiding the development of learners’ information literacy. This result may be related to the requirements brought about by new technology to manage updated copyrights and other data security regulations, which may not yet have been adopted into practice. The COVID-19 circumstances might have caused nurse educators to focus their immediate attention on learning to deliver online teaching. Therefore, they developed a deeper understanding of the essential pedagogical actions for utilizing educational technology to implement the necessary remote lectures.^11,20,21^ This could be an explanation for a lower self-assessed level on more complex digital competence dimensions. Somewhat similar findings had been reported.^11,21,25,26^ These results may be a bit concerning, given that a substantial amount of digital information is produced and processed in modern healthcare education and practice. Whether it is educators’ learning materials, students’ assignments and grades, or patients’ electronic documents, this information must be created, protected, and shared safely and ethically.^3^

Because the competence level of educators in the participating countries was consistently lower in acting safely and responsibly, targeting the planning and development of continuous education and addressing these issues as a priority could be justifiable. For example, online courses, practical workshops, and simulation education could be developed in interprofessional cooperation for implementing evidence-based knowledge for safe and ethical pedagogical approaches.^12,21,24^ Online course solutions could take the form of targeted learning pathways, such as microcredentials, conducted through short, flexible, self-paced online modules,^34^ focusing on copyright, data protection, and GDPR application, to strengthen legal awareness.^16,47^ Case-based workshops, involving interactive group work, could foster critical thinking and ethical reasoning,^13,20^ for example focusing on protecting student privacy and guidelines for avoiding plagiarism.^24^ Simulation education, in turn, would offer educators the opportunity to practice secure digital behavior (e.g. managing student data or safe communication)^16^ in risk-free settings, enabling transfer of the newly learned competence to actual practice.^4,13^ These approaches to improving competence in acting safely and responsibly could also be an area for attention in attempts to harmonize the continuous education of nurse educators internationally.^33,35^ For example, microcredential courses offer an opportunity to implement focused continuous education for professional development, which can flexibly support the development of identified competence needs in organizations.^34^

It should also be noted from this study's results that some educators (14%) self-assessed that they poorly use technology to support learners when self-directing their learning. Also, 22% of educators rarely use learning analytics to assess the progress of their students’ learning. The lack of educators’ and educational organizations’ resources may explain these results. Educators may not have had enough time for continuous education to practice the use of learning analytics (e.g. to practice student guidance based on study activity data from learning environments).^18^ On the other hand, organizations may not have had suitable learning analytics processing methods available to handle the large amounts of data available.^2^ These actions could be developed, and it is worth highlighting that students’ self-directed, meaningful, independent learning can be supported and made possible by utilizing technology in learning environments.^2,7^ For example, implementing practices that enable the scheduling of their own studies allows them to repeat or focus on more difficult subjects according to their learning needs.^7^ Additionally, nurse educators could be offered resources, such as protected time for their continuous education, to better learn how to gather learning analytics and utilize this evidence in student guidance to enhance learning progress.^2,18^ In addition, it is possible to see AI technology's potential in supporting nursing education and student learning. For example, AI helps in personalizing learning by providing each student with tailored instruction, which can enhance educational outcomes.^2,9,19^ However, further research is needed on these technologies’ reliable and ethical inclusion in nursing education.^19^

When making country comparisons, interesting differences were found in the assessments of digital competence between nurse educators from different countries. Slovak nurse educators rated their competence level higher than their Finnish, Spanish, and Maltese colleagues. Previously, only a limited comparison had been made, but no differences had been observed.^26,27^ Most likely, the observed differences in competence in this study are related to variations between the countries in educator education requirements and education conditions.^35^ Because there is no uniform educational path or educational requirements for nurse educators across Finland, Spain, Slovakia, and Malta, the competences acquired in education may vary.^35^ In addition, continuous education and its content vary from country to country.^33^ Differences in digital competence may be partly attributed to how various educator education programs prioritize evidence-based pedagogical approaches and provide opportunities to engage with educational technologies.^2,4,48^ Also, the education context and hence role of the nurse educator varies from country to country, even within an individual degree program. The lower level of competence may be explained by the research-oriented tasks of educators, in contrast to the teaching focus on contact teaching in skill labs,^35^ whereas other roles emphasize remote and online teaching.^5^ Therefore, the variability in their roles shapes differences in their perspectives on utilizing educational technology.^6^

On the other hand, variations in educator education requirements might also play a role in the differences in perceived competence. Finnish nurse educators generally have a broad range of pedagogical studies whereas Maltese educators may have a previous degree in pedagogy,^35^ which may allow them to better recognize the competence requirements for mastering educational technology in a pedagogically appropriate manner^31^—while at the same time making them more self-critical of their own competence. Therefore, when interpreting cross-national differences in educators’ competence, it is essential to critically consider the role of cultural variation in self-assessment styles. For example, Ross et al.^49^ demonstrated that Canadian university students in psychology courses showed a clearly greater tendency toward self-enhancement whereas Japanese students were even more critical in their self-assessments. Such cultural biases may confound comparative analyses of educators’ self-assessment data across the countries included in this study. In the future, more comprehensive research is needed to find out how nurse educators utilize educational technology in real-world settings as opposed to relying on self-reporting or examining how educational technology has been included in educators’ educational development to be able to teach with technology.

When examining the educators’ background variables and self-assessed digital competence level, nurse educators’ interest in technology seemed related to higher self-assessed digital competence levels. There have been suggestions that a positive attitude toward technology has increased the use of technology in teaching,^14^ and possibly, with the versatile use of technology, one's competence is perceived as higher. Therefore, it may be reasonable to encourage the utilization of educational technology in nursing education work communities. For example, it might be possible through peer mentoring for the more confident and competent nurse educators to share and demonstrate their meaningful experiences with their less confident and competent peers. This could promote a positive attitude toward the more diverse use of technology among nurse educators who are less interested in educational technology.^4,33^

Moreover, from the perspective of the development of educators’ basic and continuous education, an important result of this study is a positive relationship between a higher level of education (university degree) and completed pedagogical studies for self-assessed level of digital competence. This finding is similar to those arising from the examination of nurse educators’ competence more broadly, in which a higher level of education and completed pedagogical studies has had a positive relationship with higher self-assessed competence.^31^ This connection may occur because pedagogical studies provide theoretical and practical foundations for effective teaching. Through these studies, nurse educators have had the opportunity to enhance their competence in planning, implementing, and assessing education in line with learners’ individual needs, used learning environments, and methods. Moreover, educators may have learned to better foster self-directed learning and critical thinking and to recognize factors influencing learning, such as prior knowledge, motivation, and cultural background. Additionally, pedagogical studies help educators reflect on their professional role and build meaningful educator–student relationships.^30,32,48^ Teaching practice, as part of pedagogical studies, also may have enhanced educators’ competence in including theory in authentic practical settings. During these studies, educators also receive feedback and can reflect on their own competence in several requirements. With this pedagogical expertise acquired through broader education, educators have potentially acquired more evidence-based and versatile abilities for utilizing technology in teaching, learning, and assessment,^32,35^ which could explain the observed relationship in this study.

However, when interpreting and applying the results of this study, it should be noted that the term “pedagogical studies” lacks a clear universal operational definition. Pedagogy as a university discipline encompasses a wide range of theoretical and practical components, formed by organizational, cultural, and historical contexts. The nature, content, and structure of pedagogical education vary significantly across countries and institutions, ranging from short-term workshops to comprehensive degree programs.^48^ Based on the results, it is necessary to examine further the structure, content, and implementation of nurse educators’ education and pedagogical studies that seem to support higher levels of digital competence.

Finally, the nurse educators’ age in this study was not related to the assessed level of digital competence, in contrast to some previous studies.^6,23,26,28^ Also, in Lee and Bello's^27^ study, age was not connected to the readiness to implement online teaching. The educators’ age may not be so relevant in the examination of the level of competence because everyone has had to acquire the ability to use technology in a digitalized education system.^5,13^ In addition, during the pandemic, educators had to learn to use technology in teaching and learning.^4,21^ Also, educators have participated in continuous education to update their competence.^23^ These events and actions may have harmonized the educators’ competence levels across age groups.

When interpreting nurse educators’ digital competence level and related variables, it is also worth noting the cognitive bias that may occur in self-assessment. For example, a psychological phenomenon, the Dunning–Kruger effect, may distort self-assessment. In such a case, individuals with low competence tend to significantly overestimate their abilities whereas highly competent individuals assess their abilities more accurately and in some cases even underestimate them.^50^ In this study, this cognitive bias may have been strengthened because there are currently no objective criteria for nurse educators’ digital competence.^14,51^ Due to these constraints, potential differences between the countries and groups may remain unobserved after this study—especially when it is recognized that research on nurse educators’ digital competence level is limited.^14^ Therefore, and as stated before,^6^ versatile research is needed to conclude continuous education implementation with specific groups.

### Strengths and limitations

The study's strengths are that it produced new comparative knowledge from four European countries on a narrowly studied but important topic. Given the limited work in the field, the data collection utilized a widely used DigCompEdu framework.^16,17^ Additionally, the study was conducted collaboratively as part of a European research and development project. The STROBE checklist was used to improve the study's reporting quality.^52^

This study has some limitations. First, the response rate was 25% (n = 290), a typical response rate for an online survey of nurse education organizations.^53^ The data collection in the New Nurse Educator project did not occur within the planned scope. Despite three reminders, we did not receive the targeted number of responses, and a few organizations withdrew from the study due to scheduling challenges. However, the author team, including a statistician, considered the data sufficient for statistical analysis with the methods used in this substudy from the entire project dataset. The test results were statistically significant, but the effect sizes were weak (Kruskal–Wallis)^54,55^ or small (one-way ANOVA).^56^ Although the proportion of explained variance of the models was 28% to 32%, this is typical for social sciences and does not influence the estimated associations’ reliability.^57^ In this study, participating nurse educators responded from several organizations from four European countries. Nevertheless, the results’ generalizability should be viewed cautiously due to the relatively low number of respondents and the variation in the sizes of the participating countries. Therefore, selection and nonresponse biases should be considered when assessing the representativeness of the sample.^41^

Second, the OODI was used for the first time in Malta, Slovakia, and Spain. This was considered so that this study was part of an Erasmus + funded the New Nurse Educator project and so the research group had representation from all four countries. The OODI instrument was preliminary validated based on data from Finnish nurse educators.^36^ In this study, the research group evaluated the instrument as suitable for the context of nursing and higher education in Malta, Slovakia, and Spain. In addition, language translation, back-translation, and pretesting were performed.^38^ The alpha and omega reliability coefficients showed good reliability for the OODI (for total instrument: α = 0.949, ω = 0.954; Factor 1: α = 0.905, ω = 0.893; Factor 2: α = 0.903, ω = 0.885; Factor 3: α = 0.893, ω = 0.890).^37^ In the future, the OODI could be further validated in other new contexts, especially as educational technology continues to develop internationally. Additionally, to further develop the background variable questions of the OODI, the question about the number of completed pedagogical studies was formulated as an open-ended response. The responses were very heterogeneous (e.g. study weeks, credits, or number of courses) and could not be reported accurately.

Third, the results are based on the nurse educators’ self-assessments. Even though self-assessment in this study produced new understanding^51^ of the nurse educators’ perceived digital competence, the previously discussed cultural aspects^49^ or cognitive bias^50^ may hinder the self-assessment's accuracy.^51^ Therefore, findings may differ from actual competence and practice. It is also worth noting that this study showed indications of differences in digital competence between countries, which were not clear enough based on the effect size.^54–56^ Further research, mainly objective and observational, is needed to capture and understand how nurse educators use technology daily.^51^

## Conclusions

To conclude, nurse educators in Finland, Malta, Slovakia, and Spain report moderate digital competence. This study has increased understanding that, in all four countries, nurse educators self-assessed the safe and responsible use of technology as their weakest area of ​​digital competence. In the short term, dedicated resources need to target improving nurse educators’ continuous education, such as workshops, which could focus on supporting students’ responsible digital content creation and data protection. This study also showed differences in self-assessed digital competence; Slovak educators reported higher digital competence than educators from Finland, Malta, and Spain. Considering the limitations of self-assessment, validation of these results requires further comprehensive international research on nurse educators’ digital competence, such as research on the actions that can promote digital pedagogical practices to enhance teaching, learning, and assessment. Completing a master's or doctoral degree and pedagogical studies seems to be related to a higher level of digital competence. Therefore, consideration could be given to including pedagogical studies in nurse educators’ education. Furthermore, to support policymaking related to the organization of nurse education, a more in-depth examination of the relationship between nurse educators’ background variables and their digital competence, particularly their educational attainment and the nature of their education, is needed.

## Supplemental Material

sj-docx-1-dhj-10.1177_20552076251395451 - Supplemental material for Self-assessed digital competence of nurse educators—A cross-sectional study in four countries Supplemental material, sj-docx-1-dhj-10.1177_20552076251395451 for Self-assessed digital competence of nurse educators—A cross-sectional study in four countries by Juha Pajari, Marjorita Sormunen, Leena Salminen, Imane Elonen, Miko Pasanen, Michelle Camilleri, Laia Wennberg-Capellades, Elaine Haycock-Stuart, Andrea Sollárová and Terhi Saaranen in DIGITAL HEALTH

sj-docx-2-dhj-10.1177_20552076251395451 - Supplemental material for Self-assessed digital competence of nurse educators—A cross-sectional study in four countries Supplemental material, sj-docx-2-dhj-10.1177_20552076251395451 for Self-assessed digital competence of nurse educators—A cross-sectional study in four countries by Juha Pajari, Marjorita Sormunen, Leena Salminen, Imane Elonen, Miko Pasanen, Michelle Camilleri, Laia Wennberg-Capellades, Elaine Haycock-Stuart, Andrea Sollárová and Terhi Saaranen in DIGITAL HEALTH

sj-docx-3-dhj-10.1177_20552076251395451 - Supplemental material for Self-assessed digital competence of nurse educators—A cross-sectional study in four countries Supplemental material, sj-docx-3-dhj-10.1177_20552076251395451 for Self-assessed digital competence of nurse educators—A cross-sectional study in four countries by Juha Pajari, Marjorita Sormunen, Leena Salminen, Imane Elonen, Miko Pasanen, Michelle Camilleri, Laia Wennberg-Capellades, Elaine Haycock-Stuart, Andrea Sollárová and Terhi Saaranen in DIGITAL HEALTH

sj-docx-4-dhj-10.1177_20552076251395451 - Supplemental material for Self-assessed digital competence of nurse educators—A cross-sectional study in four countries Supplemental material, sj-docx-4-dhj-10.1177_20552076251395451 for Self-assessed digital competence of nurse educators—A cross-sectional study in four countries by Juha Pajari, Marjorita Sormunen, Leena Salminen, Imane Elonen, Miko Pasanen, Michelle Camilleri, Laia Wennberg-Capellades, Elaine Haycock-Stuart, Andrea Sollárová and Terhi Saaranen in DIGITAL HEALTH
