# Supplementary material for: Self-assessed digital competence of nurse educators—A cross-sectional study in four countries
Source: Digit Health. 2025 Nov 10;11:20552076251395451. doi: 10.1177/20552076251395451 (PMC12868590; doi:10.1177/20552076251395451)
Supplement: sj-docx-2-dhj-10.1177_20552076251395451 - Supplemental material for Self-assessed digital competence of nurse educators—A cross-sectional study in four countries [file sj-docx-2-dhj-10.1177_20552076251395451.docx]

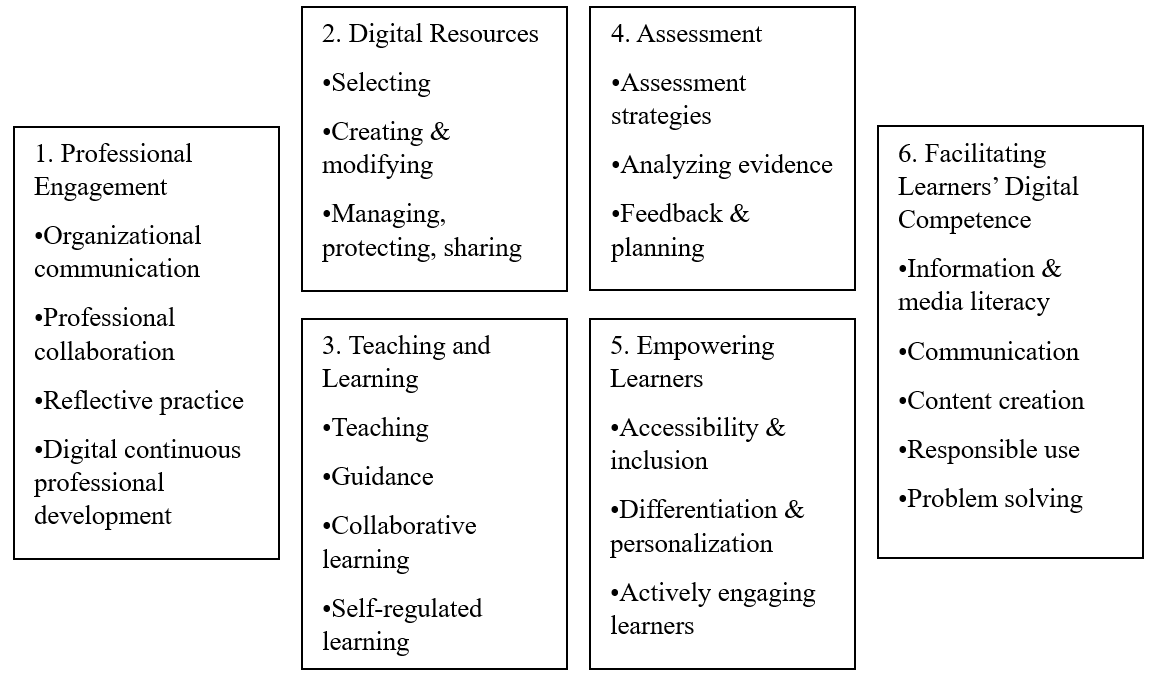


Supplementary Digital Content 1, Figure 1. The European Framework for the Digital Competence of Educators (DigCompEdu) According to the Publication of the European Commission^16^
