# Supplementary material for: Self-assessed digital competence of nurse educators—A cross-sectional study in four countries
Source: Digit Health. 2025 Nov 10;11:20552076251395451. doi: 10.1177/20552076251395451 (PMC12868590; doi:10.1177/20552076251395451)
Supplement: sj-docx-3-dhj-10.1177_20552076251395451 - Supplemental material for Self-assessed digital competence of nurse educators—A cross-sectional study in four countries [file sj-docx-3-dhj-10.1177_20552076251395451.docx]

Supplementary Digital Content 2, Table 1. Nurse Educators Background Information

| **Background information** | **n** | **%** | **Mean** | **SD** | **Min** | **Max** |
| --- | --- | --- | --- | --- | --- | --- |
| **Age** (n = 284) |  |  | 47.99 | 9.21 | 24 | 67 |
| **Highest level of education** (n = 290) |  |  |  |  |  |  |
| University degree ^a^ | 266 | 92 |  |  |  |  |
| Other degree ^b^ | 24 | 8 |  |  |  |  |
| **Clinical work experience** (n = 281) |  |  | 15.53 | 9.72 | 0 | 42 |
| ≤5 | 43 | 15 |  |  |  |  |
| 6–10 | 77 | 27 |  |  |  |  |
| 11–15 | 37 | 13 |  |  |  |  |
| 16–20 | 49 | 18 |  |  |  |  |
| >20 | 75 | 27 |  |  |  |  |
| **Pedagogical studies** (n = 289) |  |  |  |  |  |  |
| Yes | 212 | 73 |  |  |  |  |
| No | 77 | 27 |  |  |  |  |
| **Participation continuous professional education** (n = 284) |  |  |  |  |  |  |
| Yes | 243 | 82 |  |  |  |  |
| No | 50 | 18 |  |  |  |  |
| **Work experience as an educator** (n = 288) |  |  | 12.66 | 9.39 | 0 | 45 |
| ≤5 | 81 | 28 |  |  |  |  |
| 6–10 | 55 | 19 |  |  |  |  |
| 11–15 | 63 | 22 |  |  |  |  |
| 16–20 | 31 | 11 |  |  |  |  |
| >20 | 58 | 20 |  |  |  |  |

a = A doctorate or master’s degree at a university, b = A master’s degree at a university of applied sciences, a university of applied sciences degree, or a lower level of education
