# Supplementary material for: Self-assessed digital competence of nurse educators—A cross-sectional study in four countries
Source: Digit Health. 2025 Nov 10;11:20552076251395451. doi: 10.1177/20552076251395451 (PMC12868590; doi:10.1177/20552076251395451)
Supplement: sj-docx-4-dhj-10.1177_20552076251395451 - Supplemental material for Self-assessed digital competence of nurse educators—A cross-sectional study in four countries [file sj-docx-4-dhj-10.1177_20552076251395451.docx]

Supplementary Digital Content 3, Table 2. Digital Competence of Nurse Educators with Descriptive Statistical Results of the OODI Instrument

| OODI Items | | Value (1 - 5) * | | | | |
| --- | --- | --- | --- | --- | --- | --- |
|  |  | 1 | 2 | 3 | 4 | 5 |
|  | Country (n) | f (%) | f (%) | f (%) | f (%) | f (%) |
| **Factor 1, Implementing appropriate independent and community learning** | | | | | | |
| Using technology to collaborate with colleagues, students, and other partners. | | | | | | |
|  | Finland (110) | 0 (0) | 0 (0) | 9 (8) | 57 (52) | 44 (40) |
|  | Malta (19) | 0 (0) | 2 (10) | 1 (5) | 10 (53) | 6 (32) |
|  | Spain (107) | 0 (0) | 4 (4) | 10 (9) | 46 (43) | 47 (44) |
|  | Slovakia (51) | 0 (0) | 2 (4) | 1 (2) | 28 (55) | 20 (39) |
|  | All (287) | 0 (0) | 8 (3) | 21 (7) | 141 (49) | 117 (41) |
| Using digital resources to support my continuous professional development. | | | | | | |
|  | Finland (110) | 1 (1) | 8 (7) | 27 (25) | 54 (49) | 20 (18) |
|  | Malta (20) | 0 (0) | 1 (5) | 7 (35) | 8 (40) | 4 (20) |
|  | Spain (108) | 0 (0) | 2 (2) | 19 (17) | 57 (53) | 30 (28) |
|  | Slovakia (51) | 2 (4) | 3 (6) | 7 (14) | 23 (45) | 16 (31) |
|  | All (289) | 3 (1) | 14 (5) | 60 (21) | 142 (49) | 70 (24) |
| Evaluating of own digital competence. | | | | | | |
|  | Finland (111) | 0 (0) | 3 (3) | 14 (12) | 64 (58) | 30 (27) |
|  | Malta (20) | 0 (0) | 2 (10) | 8 (40) | 9 (45) | 1 (5) |
|  | Spain (108) | 2 (2) | 10 (9) | 26 (24) | 52 (48) | 18 (17) |
|  | Slovakia (51) | 1 (2) | 5 (10) | 10 (20) | 25 (48) | 10 (20) |
|  | All (290) | 3 (1) | 20 (7) | 58 (20) | 150 (52) | 59 (20) |
| Using technology to guide learners. | | | | | | |
|  | Finland (111) | 0 (0) | 4 (4) | 19 (17) | 64 (58) | 24 (21) |
|  | Malta (20) | 0 (0) | 2 (10) | 8 (40) | 6 (30) | 5 (20) |
|  | Spain (107) | 1 (1) | 7 (6) | 33 (31) | 48 (45) | 18 (17) |
|  | Slovakia (50) | 1 (2) | 1 (2) | 11 (22) | 25 (50) | 12 (24) |
|  | All (288) | 2 (1) | 14 (5) | 71 (25) | 143 (49) | 58 (20) |
| When choosing digital resources, considering their suitability for teaching and learning. | | | | | | |
|  | Finland (110) | 0 (0) | 4 (4) | 30 (27) | 64 (58) | 12 (11) |
|  | Malta (18) | 0 (0) | 1 (6) | 6 (33) | 11 (61) | 0 (0) |
|  | Spain (108) | 0 (0) | 6 (6) | 35 (32) | 52 (48) | 15 (14) |
|  | Slovakia (49) | 0 (0) | 1 (2) | 10 (20) | 26 (53) | 12 (25) |
|  | All (285) | 0 (0) | 12 (4) | 81 (28) | 153 (54) | 39 (14) |
| Producing digital learning materials, considering their suitability for teaching and learning. | | | | | | |
|  | Finland (111) | 0 (0) | 10 (9) | 39 (35) | 49 (44) | 13 (12) |
|  | Malta (20) | 0 (0) | 4 (20) | 3 (15) | 11 (55) | 2 (10) |
|  | Spain (108) | 1 (1) | 12 (11) | 28 (26) | 48 (44) | 19 (18) |
|  | Slovakia (49) | 0 (0) | 3 (6) | 12 (25) | 24 (49) | 10 (20) |
|  | All (288) | 1 (1) | 29 (10) | 82 (28) | 132 (46) | 44 (15) |
| Using technology in teaching to enable collaborative learning. | | | | | | |
|  | Finland (111) | 1 (1) | 8 (7) | 34 (30) | 54 (49) | 14 (13) |
|  | Malta (20) | 0 (0) | 2 (10) | 8 (40) | 7 (35) | 3 (15) |
|  | Spain (108) | 1 (1) | 8 (7) | 40 (37) | 45 (42) | 14 (13) |
|  | Slovakia (50) | 0 (0) | 2 (4) | 13 (26) | 29 (58) | 6 (12) |
|  | All (289) | 2 (1) | 20 (7) | 95 (33) | 135 (46) | 37 (13) |
| Supporting learners' self-direction through technology. | | | | | | |
|  | Finland (109) | 0 (0) | 10 (9) | 38 (35) | 48 (44) | 13 (12) |
|  | Malta (20) | 0 (0) | 5 (25) | 5 (25) | 6 (30) | 4 (20) |
|  | Spain (106) | 1 (1) | 18 (17) | 36 (34) | 42 (40) | 9 (8) |
|  | Slovakia (50) | 0 (0) | 4 (8) | 17 (34) | 23 (46) | 6 (12) |
|  | All (285) | 1 (1) | 37 (13) | 96 (33) | 119 (42) | 32 (11) |
| **Factor 2, Acting safely and responsibly** | | | | | | |
| Protecting the privacy of sensitive digital material used in teaching. | | | | | | |
|  | Finland (111) | 7 (6) | 19 (17) | 32 (29) | 38 (34) | 15 (14) |
|  | Malta (20) | 0 (0) | 2 (10) | 5 (25) | 9 (45) | 4 (20) |
|  | Spain (108) | 1 (1) | 15 (14) | 21 (19) | 42 (39) | 29 (27) |
|  | Slovakia (50) | 1 (2) | 4 (8) | 8 (16) | 21 (42) | 16 (32) |
|  | All (289) | 9 (3) | 40 (14) | 66 (23) | 110 (38) | 64 (22) |
| Using digitally licensed digital materials in the right way. | | | | | | |
|  | Finland (111) | 4 (4) | 16 (14) | 30 (27) | 48 (43) | 13 (12) |
|  | Malta (20) | 1 (5) | 1 (5) | 5 (25) | 11 (55) | 2 (10) |
|  | Spain (108) | 5 (5) | 13 (12) | 26 (24) | 41 (38) | 23 (21) |
|  | Slovakia (50) | 2 (4) | 3 (6) | 11 (22) | 19 (38) | 15 (30) |
|  | All (289) | 12 (4) | 33 (12) | 72 (25) | 119 (41) | 53 (18) |
| Guiding learners to the critical use of digital media. | | | | | | |
|  | Finland (110) | 1 (1) | 9 (8) | 26 (23) | 59 (54) | 15 (14) |
|  | Malta (20) | 3 (15) | 5 (25) | 6 (30) | 5 (25) | 1 (5) |
|  | Spain (108) | 0 (0) | 14 (13) | 36 (33) | 44 (41) | 14 (13) |
|  | Slovakia (50) | 1 (2) | 5 (10) | 14 (28) | 21 (42) | 9 (18) |
|  | All (288) | 5 (2) | 33 (11) | 82 (28) | 129 (45) | 39 (14) |
| Supporting learners to produce responsible digital content. | | | | | | |
|  | Finland (111) | 3 (3) | 13 (13) | 44 (39) | 39 (35) | 11 (10) |
|  | Malta (20) | 3 (15) | 2 (10) | 9 (45) | 5 (25) | 1 (5) |
|  | Spain (108) | 4 (4) | 21 (19) | 41 (38) | 32 (30) | 10 (9) |
|  | Slovakia (49) | 0 (0) | 5 (10) | 20 (41) | 16 (33) | 8 (16) |
|  | All (288) | 10 (4) | 42 (15) | 114 (39) | 92 (32) | 30 (10) |
| Guiding the development of learners' information literacy in a digital learning environment. | | | | | | |
|  | Finland (111) | 2 (2) | 13 (12) | 47 (42) | 42 (38) | 7 (6) |
|  | Malta (20) | 3 (15) | 5 (25) | 8 (40) | 3 (15) | 1 (5) |
|  | Spain (108) | 2 (2) | 20 (18) | 42 (39) | 37 (34) | 7 (7) |
|  | Slovakia (50) | 1 (2) | 8 (16) | 17 (34) | 17 (34) | 7 (14) |
|  | All (289) | 8 (3) | 46 (16) | 114 (39) | 99 (34) | 22 (8) |
| Supporting learners' ability to solve problems with technology. | | | | | | |
|  | Finland (111) | 5 (5) | 18 (16) | 49 (44) | 34 (30) | 5 (5) |
|  | Malta (20) | 3 (15) | 3 (15) | 8 (40) | 5 (25) | 1 (5) |
|  | Spain (106) | 4 (4) | 20 (19) | 45 (42) | 31 (29) | 6 (6) |
|  | Slovakia (48) | 1 (2) | 6 (13) | 21 (44) | 16 (31) | 5 (10) |
|  | All (285) | 13 (2) | 47 (16) | 123 (43) | 85 (30) | 17 (6) |
| Guiding learners to protect the material they produce in accordance with copyright law. | | | | | | |
|  | Finland (110) | 12 (11) | 26 (24) | 42 (38) | 23 (21) | 7 (6) |
|  | Malta (20) | 1 (5) | 6 (30) | 8 (40) | 4 (20) | 1 (5) |
|  | Spain (108) | 13 (12) | 30 (28) | 23 (21) | 34 (32) | 8 (7) |
|  | Slovakia (50) | 3 (6) | 2 (4) | 18 (36) | 17 (34) | 10 (20) |
|  | All (288) | 29 (10) | 64 (22) | 91 (32) | 78 (27) | 26 (9) |
| **Factor 3, Guiding learning based on the evidence** | | | | | | |
| Using technology to give feedback to learners. | | | | | | |
|  | Finland (111) | 4 (4) | 13 (12) | 33 (29) | 49 (44) | 12 (11) |
|  | Malta (20) | 1 (5) | 2 (10) | 5 (25) | 10 (50) | 2 (10) |
|  | Spain (107) | 0 (0) | 14 (13) | 36 (34) | 45 (42) | 12 (11) |
|  | Slovakia (50) | 0 (0) | 3 (6) | 16 (32) | 19 (38) | 12 (24) |
|  | All (288) | 5 (2) | 32 (11) | 90 (31) | 123 (43) | 38 (13) |
| Motivating learners in a digital environment. | | | | | | |
|  | Finland (108) | 0 (0) | 11 (10) | 41 (38) | 46 (43) | 10 (9) |
|  | Malta (20) | 1 (5) | 4 (20) | 9 (45) | 3 (15) | 3 (15) |
|  | Spain (108) | 1 (1) | 11 (10) | 42 (39) | 44 (41) | 10 (9) |
|  | Slovakia (50) | 0 (0) | 5 (10) | 11 (22) | 28 (56) | 6 (12) |
|  | All (286) | 2 (1) | 31 (11) | 103 (36) | 121 (42) | 29 (10) |
| Using technology in individualized teaching. | | | | | | |
|  | Finland (111) | 1 (1) | 20 (18) | 39 (35) | 43 (39) | 8 (7) |
|  | Malta (20) | 1 (5) | 3 (15) | 8 (40) | 6 (30) | 2 (10) |
|  | Spain (107) | 1 (1) | 6 (6) | 45 (42) | 45 (42) | 10 (9) |
|  | Slovakia (50) | 0 (0) | 3 (6) | 11 (22) | 29 (58) | 7 (14) |
|  | All (288) | 3 (1) | 32 (11) | 103 (36) | 123 (43) | 27 (9) |
| Using technology to assess learning according to different assessment methods. | | | | | | |
|  | Finland (109) | 2 (2) | 12 (11) | 40 (37) | 43 (39) | 12 (11) |
|  | Malta (20) | 0 (0) | 4 (20) | 7 (35) | 6 (30) | 3 (15) |
|  | Spain (106) | 1 (1) | 11 (10) | 46 (43) | 39 (37) | 9 (9) |
|  | Slovakia (50) | 0 (0) | 5 (10) | 16 (32) | 21 (42) | 8 (16) |
|  | All (285) | 3 (1) | 32 (11) | 109 (38) | 109 (38) | 32 (11) |
| Using learning analytics to assess the progress of learning. | | | | | | |
|  | Finland (111) | 8 (7) | 26 (24) | 44 (40) | 26 (23) | 7 (6) |
|  | Malta (20) | 1 (5) | 5 (25) | 8 (40) | 4 (20) | 2 (10) |
|  | Spain (107) | 2 (2) | 16 (15) | 48 (45) | 32 (30) | 9 (8) |
|  | Slovakia (50) | 0 (0) | 4 (8) | 17 (34) | 19 (38) | 10 (20) |
|  | All (288) | 11 (4) | 51 (18) | 117 (40) | 81 (28) | 28 (10) |

* 1 = Not at all, 2 = Poorly, 3 = Neither poorly nor well, 4 = Well, 5 = Very well
